# PP25 Publication Of Health Technology Assessments In The “Epidemiology and Health Services: Journal Of The Brazilian National Health System” – A Cluster Analysis

**DOI:** 10.1017/S0266462325102444

**Published:** 2025-12-29

**Authors:** Tais Galvao, Marcus T. Silva

## Abstract

**Introduction:**

The Epidemiology and Health Services: Journal of the Brazilian National Health System (RESS; Epidemiologia e Serviços de Saúde: revista do SUS) is a journal edited by the Brazilian Ministry of Health focused on disseminating scientific evidence relevant to the Brazilian Unified Health System. We aimed to assess the patterns of publication relevant to health technology assessment (HTA) and appraise the journal’s contribution to advancing knowledge and decision-making in the field.

**Methods:**

We performed a cluster analysis of RESS publications from 2016 to 2024. Abstracts were imported from PubMed to EndNote and exported to Excel. Text preprocessing (tokenization, removal of punctuation, numbers, stop words, and stemming) was performed. Abstracts with terms related to diseases and technologies were regarded as HTA relevant. Corpuses were converted into term frequency–inverse document frequency, and dimensionality was reduced using principal component analysis. K-means clustering with silhouette coefficient for consistency were calculated. Thematic groups were analyzed based on frequent terms, document distribution, and intra-cluster similarity and compared to HTA Core Model®. Python 3.13 was used for analyses.

**Results:**

From 674 abstracts, 409 were deemed relevant for HTA. Six clusters were identified: risk factors (22.2% of abstracts; cohesion 95.5%), health services (33.5%; cohesion 0.8%), infections and clinical services (18.8%; cohesion 86.4%), mortality trends (14.2%; cohesion 95.6%), causes of death (8.8%; cohesion 86.9%), and congenital syphilis (2.4%; cohesion 94.2%). The silhouette coefficient was 0.4, indicating moderate clustering quality. Most clusters displayed high internal cohesion, except for health services, and were related to HTA domains of current use, safety, organization, and patient and social. No clusterization of technical, clinical effectiveness, cost and economic, ethical analysis, or legal domains was observed.

**Conclusions:**

Main patterns of HTA relevant research were identified, with thematic clusters such as risk factors, mortality, health services, and infections. Other HTA relevant domains—like economic evaluations and patient-reported outcomes—were not detected in the clusters, suggesting a gap in RESS focus. To improve its relevance for the Brazilian HTA field, RESS should prioritize less represented HTA domains.

